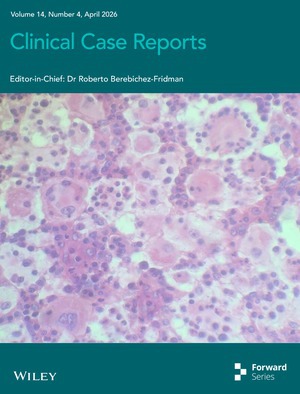# Cover Image

**DOI:** 10.1002/ccr3.72542

**Published:** 2026-04-09

**Authors:** George Evele, Kouya Francine, Richard Bardin

## Abstract

The cover image is based on the article *Clinicopathological Features, Treatment Response, and Outcome of Rosai‐Dorfman Disease in Two Children* by George Evele et al., https://doi.org/10.1002/ccr3.72471.